# Evidence for a schedule-dependent deleterious interaction between paclitaxel, vinblastine and cisplatin (PVC) in the treatment of advanced transitional cell carcinoma

**DOI:** 10.1054/bjoc.2000.1480

**Published:** 2000-12

**Authors:** C Mulatero, B R McClaren, M Mason, R T D Oliver, C J Gallagher

**Affiliations:** Dept. of Medical Oncology, St Bartholomew's Hospital, London; Dept. of Clinical Oncology, Velindre Hospital, Cardiff, UK

**Keywords:** transitional cell, paclitaxel, vinblastine, cisplatin, schedule

## Abstract

A phase II study to evaluate the efficacy and toxicity of the combination of vinblastine, paclitaxel and cisplatin (PVC) in previously untreated patients with advanced transitional cell carcinoma. Chemotherapy naive patients with locally advanced or metastatic transitional cell carcinoma received the intravenous combination of paclitaxel 175 mg/m^2^ over three hours followed by cisplatin 70 mg/m^2^ over 3 hours on day 1 and vinblastine 3 mg/m^2^ as a bolus on days 1 and 8 on a 21-day cycle, to a maximum of 6 cycles. The day 8 vinblastine was omitted if the total neutrophil count was <1.0. 15 patients (13 M, 2 F) of median age 66 (54–75) received a median of 5 cycles of treatment. There were two complete responses (13%; 95% CI 2–40%) and five partial responses (33%; 95% CI 12–62%), for an overall response rate of 46% (95% CI 21–73%). Responses occurred only in those with locally recurrent tumours and/or lymph nodes involved. Neutropenia at Grade 3–4 occurred in 14 of 67 cycles (21%) resulting in 7 episodes of neutropenic sepsis. Grade 3–4 thrombocytopenia was not observed. Other Grade 3 toxicity included alopecia (10 pts), diarrhoea (2 pts), constipation resulting in bowel obstruction (2 pts), nephrotoxicity (1 pt), myalgic pain (1 pt) and peripheral neuropathy (1 pt). Six patients developed Grade 2 paraesthesia. The median time to progression was 6 months and the median survival was 11 months. The regimen PVC was both less effective against transitional cell carcinoma and less toxic than expected. This may reflect an inhibitory interaction between vinblastine and paclitaxel and this schedule cannot be recommended for further investigation. © 2000 Cancer Research Campaign http://www.bjcancer.com


*British Journal of Cancer* (2000) **83**(12), 1612–1616
© 2000 Cancer Research Campaign
doi: 10.1054/ bjoc.2000.1480, available online at http://www.idealibrary.com on IDEAL®

http://www.bjcancer.com

### Evidence for a schedule-dependent deleterious interaction between paclitaxel, vinblastine and cisplatin (PVC) in the treatment of advanced transitional cell carcinoma

**C Mulatero[1], BR McClaren[1], M Mason[2], RTD Oliver[1] and CJ Gallagher[1]**

[1]Dept. of Medical Oncology, St Bartholomew's Hospital, London, and [2]Dept. of Clinical Oncology, Velindre Hospital, Cardiff, UK

**Summary** A phase II study to evaluate the efficacy and toxicity of the combination of vinblastine, paclitaxel and cisplatin (PVC) in previously untreated patients with advanced transitional cell carcinoma. Chemotherapy naive patients with locally advanced or metastatic transitional cell carcinoma received the intravenous combination of paclitaxel 175 mg/m$^2$ over three hours followed by cisplatin 70 mg/m$^2$ over 3 hours on day 1 and vinblastine 3 mg/m$^2$ as a bolus on days 1 and 8 on a 21-day cycle, to a maximum of 6 cycles. The day 8 vinblastine was omitted if the total neutrophil count was <1.0. 15 patients (13 M, 2 F) of median age 66 (54–75) received a median of 5 cycles of treatment. There were two complete responses (13%; 95% CI 2–40%) and five partial responses (33%; 95% CI 12–62%), for an overall response rate of 46% (95% CI 21–73%). Responses occurred only in those with locally recurrent tumours and/or lymph nodes involved. Neutropenia at Grade 3–4 occurred in 14 of 67 cycles (21%) resulting in 7 episodes of neutropenic sepsis. Grade 3–4 thrombocytopenia was not observed. Other Grade 3 toxicity included alopecia (10 pts), diarrhoea (2 pts), constipation resulting in bowel obstruction (2 pts), nephrotoxicity (1 pt), myalgic pain (1 pt) and peripheral neuropathy (1 pt). Six patients developed Grade 2 paraesthesia. The median time to progression was 6 months and the median survival was 11 months. The regimen PVC was both less effective against transitional cell carcinoma and less toxic than expected. This may reflect an inhibitory interaction between vinblastine and paclitaxel and this schedule cannot be recommended for further investigation. © 2000 Cancer Research Campaign http://www.bjcancer.com

**Keywords:** transitional cell; paclitaxel; vinblastine; cisplatin; schedule

Bladder cancer is the sixth most common cancer, worldwide. Approximately 54 000 new cases are diagnosed and over 12 000 deaths result from it annually in the USA (Parker et al, 1997). The median survival for untreated invasive bladder cancer is 6 to 9 months for those with distant metastatic disease and 18 months for those with locally advanced disease and/or involved regional lymph nodes. The most active conventional single agents include cisplatin (Yagoda et al, 1976; Merrin, 1978; Rossof et al, 1979; Herr, 1980; Peters and MR, 1980; Oliver et al, 1981), methotrexate (Natale et al, 1981; Oliver et al, 1984), vinblastine (Blumenreich et al, 1982) and doxorubicin (Yagoda et al, 1977). Pooled data from Phase II studies indicate a response rate of 30% for cisplatin (95% CI 25–35%) and 29% for methotrexate (95% CI 23–35%) (Yagoda, 1987). The M-VAC regimen containing methotrexate, vinblastine, doxorubicin and cisplatin which was first reported by Sternberg et al provided the first real improvement over single agent treatment (Sternberg et al, 1985). The long-term follow-up data on 203 patients who underwent treatment with the M-VAC regimen has recently been updated. An overall response (OR) rate of 67% was observed with a complete response (CR) rate of 23% (Bajorin et al, 1998a). The median overall survival (OS) of the whole group was 14.3 months. In a multi-centre phase III trial the Intergroup compared M-VAC versus cisplatin. They found that M-VAC was superior but at the expense of increased toxicity with significant thrombocytopenia, and leukocytopenia of Grade 3 or 4 in 21% and 58%, respectively. Neutropenic sepsis occurred in 25% and was fatal in 3% (Loehrer et al, 1992).

Efforts have been made to find combinations that demonstrate better efficacy than the M-VAC regimen but with reduced toxicity. The CMV regimen consisting of cisplatin, methotrexate, and vinblastine initially reported by Harker et al was less toxic and showed OR of 56% and CR rate of 28%. However the median OS was 8 months (Harker et al, 1985). Whilst other regimens utilizing conventional agents in accelerated schedules of M-VAC or CMV have been tried with similar benefit (Boshoff et al, 1995; Dodd et al, 1998) the advent of new agents with activity in transitional cell carcinoma invite the testing of novel regimens that may improve outcomes.

Paclitaxel shows high activity as a single agent in untreated transitional cell carcinoma of the bladder. The Eastern Co-operative Oncology Group demonstrated a 42% response rate in a Phase II study of 26 patients (Roth et al, 1994), although in a group of 14 previously treated patients Papamichael et al found only 1 partial response for a response rate of 7% (Papamichael et al, 1997). Prompted by these observations our study substituted paclitaxel for methotrexate in the CMV regimen, investigating the combination of paclitaxel with vinblastine and cisplatin in previously untreated patients.

*Received 16 February 2000*
*Revised 25 July 2000*
*Accepted 9 August 2000*

*Correspondence to:* Dr CJ Gallagher

### PATIENTS AND METHODS

### Patient population

Eligible patients were between the ages of 18 and 75 years old with histologically confirmed advanced transitional cell carcinoma of the urothelium, bi-dimensionally measurable on physical examination, chest X-ray or computed tomography (CT) examination. Patients had not received prior systemic chemotherapy nor had radiotherapy in the preceding four weeks and required an ECOG score of ≤2. A glomerular filtration rate >50 ml $min^{-1}$ measured by urinary creatinine clearance or EDTA clearance, and a serum bilirubin level <25 mmol/l, WBC >3000 $\mu l^{-1}$ and platelet count >100 000 $\mu l^{-1}$ were required. Patients with a history of prior malignancy, except basal cell or squamous cell carcinoma of the skin or in situ carcinoma of the cervix were excluded from the study. Those patients with New York Heart Association Functional Classification >1 were considered ineligible, and were those with pre-existing peripheral neuropathy of WHO grade >1 or those with active infection or other serious underlying medical condition including prior allergic reaction to cremophor with cyclosporin or vitamin K. Local Research Ethics Committee approval was obtained and all participants gave written informed consent.

### Baseline data

Pretreatment investigations included a physical examination, blood investigations including full blood count (FBC), erythrocyte sedimentation rate (ESR), renal and liver function test and serum lactate dehydrogenase (LDH) and β human chorionic-gonadotrophin (βhCG). A urinary 24-hour creatinine clearance or EDTA was performed. Imaging investigations included a chest radiograph and a CT of the abdomen and pelvis. A radionuclide bone scan was performed only if clinically indicated or if serum alkaline phosphatase (ALP) was elevated. In patients with locally advanced disease cystoscopy and biopsy together with urine cytology was performed.

### Therapy

Vinblastine at a dose of 3 $mg/m^2$ was administered as an intravenous bolus injection on days 1 and 8, followed on day 1 by paclitaxel 175 $mg/m^2$ in 500 ml of dextrose or 0.9% sodium chloride solution as an intravenous infusion over 3 hours. All patients received dexamethasone 20 mg orally 12 and 6 hours before the paclitaxel and chlorpheniramine 10 mg and cimetidine 300 mg intravenously 30 minutes before the infusion. Finally following hydration with 500 ml of 0.9% sodium chloride solution, cisplatin 70 $mg/m^2$ in 1 l 0.9% sodium chloride was administered over two hours. Thereafter a urine output of 100 $ml/h^{-1}$ was maintained with one litre of 0.9% sodium chloride solution containing 20 mmol of magnesium sulphate and 40 mmol of potassium chloride over the next 3 hours. The day 8 vinblastine dose was omitted if the absolute neutrophil count had fallen to <1000 $\mu l^{-1}$. This cycle was repeated every 21 days.

### Evaluation

Response to treatment was first assessed following a minimum of two cycles of therapy by chest radiograph or CT scan, and assessed according to UICC criteria (Hayward et al, 1977). In patients with progressive disease following 2 cycles of treatment chemotherapy was discontinued. In cases with stable disease or responses after two cycles, a total of 6 cycles of treatment was administered.

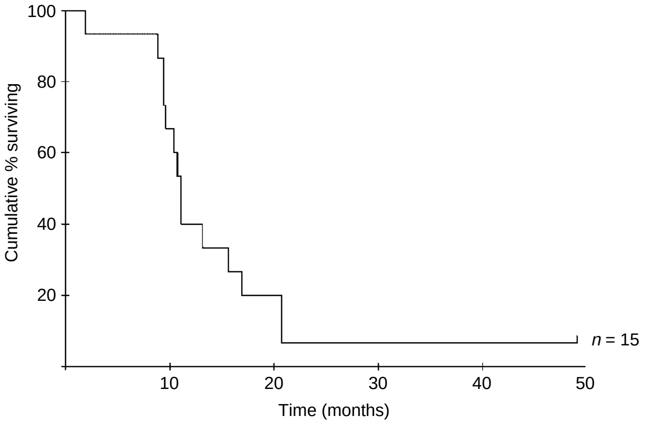

**Figure 1** Kaplan-Meier plot of overall survival

**Table 1** Patient characteristics.

| Characteristic | Number of patients |
|---|---|
| *Age years* | |
| Median | 66 |
| Range | 54–73 |
| Sex | |
| Male | 13 |
| Female | 2 |
| *Sites of metastases* | |
| Lymph nodes | 10 |
| Bladder | 6 |
| Lungs | 5 |
| Liver | 3 |
| Pelvis | 2 |
| Bone | 2 |
| Ureter | 1 |
| Prostate | 1 |

$n$ = 15

### Statistical analysis

The data were complete as of January 1999. All patients who completed day 1 of treatment were considered assessable for response and survival. Response duration and survival were measured from the date of first treatment. Overall survival was estimated by the Kaplan-Meier method and is shown in Figure 1.

### RESULTS

Between March 1995 and February 1996 15 patients were treated, of which 13 were male and two were female. The bladder was the primary site in all cases. The median age was 66 years (range 54–73). 14 patients had previously undergone surgery, 6 having had radical tumour resection (3 having undergone a cystectomy, one a nephroureterectomy, one a cystoprostatectomy and one an ileocystoplasty) and 8 having undergone transurethral resection. One patient was deemed to have inoperable metastatic disease. Five patients had relapsed following prior primary pelvic radiotherapy

© 2000 Cancer Research Campaign

**Table 2** Non-haematological toxicity.

| Non-haematological toxicity | Grade 0 | 1 | 2 | 3 | 4 |
|---|---|---|---|---|---|
| Alopecia | 1 | 1 | 3 | 10 | – |
| Peripheral neuropathy | 6 | 1 | 7 | 3 | 0 |
| Mucositis | 13 | 0 | 2 | 0 | 0 |
| Nausea and vomiting | 4 | 6 | 3 | 1 | 1 |
| Constipation | 10 | 0 | 1 | 4 | 0 |
| Infection | 2 | 5 | 1 | 5 | 0 |

*n* = 15

**Table 3** Response to treatment.

| Best response | Number | Percentage |
|---|---|---|
| CR | 2 | 13 |
| PR | 5 | 33 |
| SD | 4 | 27 |
| PD | 3 | 20 |
| NE | 1 | 7 |
| Total | 15 | 100 |

*n* = 15

for TCC of the bladder. A total of 67 cycles of treatment were administered with a median of 5 cycles per patient (range 1–6). Patient characteristics and sites of disease are summarized in Table 1.

### Toxicity

Haematological toxicity was significant with Grade 3/4 neutropenia observed in 14 of 67 (21%) cycles with 7 resulting episodes of neutropenic sepsis. The day 8 vinblastine dosage was omitted in a total of 11 (16%) cycles because of low neutrophil count. There were no episodes of thrombocytopenia greater than Grade 2.

Non-haematological toxicity of Grade 3 or 4 included alopecia in 10 patients, diarrhoea in 2 patients. Subacute bowel obstruction occurred in 2 patients on their first cycle of therapy, neither of whom received further cycles of this regimen. One patient developed Grade 3 painful myalgia. Six patients developed Grade 2 sensory neuropathy, but only one patient progressed to Grade 3. Non-haematological toxicity is summarized in Table 2.

### Response

All 15 patients are included in response calculations although it was not possible to evaluate one patient because of toxicity following cycle one. A complete remission was achieved in two patients (13%; 95% CI 2–40%) and a partial remission in 5 patients (33%; 95% CI 12–62%). The OR rate was thus 46% (95% CI 21–73%). 4 patients had SD at completion of treatment and 3 had PD. The trial was discontinued after 15 patients when it became apparent that the response rate was unlikely to be superior to that observed with the MVAC regimen. All of the responses occurred in those with locoregional disease. Response to treatment is summarized in Table 3.

The median time to progression for all patients was 6 months. Two patients subsequently received further chemotherapy, 3 received radiotherapy for pelvic disease and 2 radiotherapy at distant metastatic sites.

The median overall survival is 11 months (range 9–48 months) as of November 1999. 14 patients have died of disease (range 9–21 months) and one patient is alive with no evidence of disease progression at 48 months from therapy.

### DISCUSSION

We observed an overall response rate in this study of 46% (95% CI 21–73%) and a median survival of 15 months in patients receiving PVC for metastatic bladder TCC. Toxicity was manageable in all but two patients with extensive intra-abdominal disease who developed subacute bowel obstruction after the first cycle of treatment.

There is increasing interest in the schedule dependency of drug combinations involving paclitaxel. We adopted a schedule with vinblastine administered immediately prior to paclitaxel. Paclitaxel and vinblastine represent two classes of drug that target tubulin. They have separate binding properties and opposing mechanisms of action. Subsequently it has been shown in vitro that there is sequence dependence, with increased stability of paclitaxel-induced tubulin polymerization and concomitant increase in cytotoxicity if the paclitaxel is preceded by vinblastine by 48 hours (Giannakakou et al, 1998). However if administered simultaneously there is a diminution of the paclitaxel-induced tubulin polymerization and reduced cytotoxicity below that of single agent treatment. Our regimen involved a close temporal sequence of administration and this may have reduced the efficacy of this combination.

The choice of paclitaxel dosage and duration of infusion are also important variables in evaluating the efficacy of paclitaxel combination regimens. We chose to give a short 3-hour infusion of paclitaxel followed by cisplatin. There is debate over the optimal dosage and duration of infusion of paclitaxel in the literature. Our chosen dosage schedule of 175 $mg/m^2$ over 3 hours on a 21-day cycle has been shown to be active in combination with cisplatin in carcinoma of the ovary and is associated with minimal neurotoxicity.

A synergy has been observed between paclitaxel and cisplatin in vitro in ovarian cell lines, provided that the paclitaxel is administered prior to the cisplatin (Rowinsky et al, 1993; Jekunen et al, 1994). Preliminary Phase II data on the combination of paclitaxel and cisplatin in previously untreated patients with transitional cell carcinoma have shown higher response rates than we observed with PVC. Burch et al administered paclitaxel 135 $mg/m^2$ over 3 hours followed by cisplatin 70 $mg/m^2$ on day 1 of a 21-day cycle. They observed responses in 21 of 29 patients (OR 72%, CI 56–90%) with 10 CRs, and a median time to progression of 8 months and a median survival of 13 months (Burch et al, 1999). Murphy et al reported 13 responses in 18 evaluable patients (OR rate of 72%) using paclitaxel 170 $mg/m^2$ over 24 hours followed by cisplatin 75 $mg/m^2$ on day 1 of a 21-day cycle (Murphy et al, 1996). In a multi-institutional ECOG study a regimen using paclitaxel 225 $mg/m^2$ over 3 hours followed by cisplatin 75 $mg/m^2$ on day 1 of a 21-day cycle has been reported by Dreicer et al. They have observed 13 objective responses in 20 evaluable patients (63%, CI 42–81%) (Dreicer et al, 1998). A paclitaxel containing triple combination, based upon the CMV regimen, consisting of paclitaxel 200 $mg/m^2$ over 3 hours followed by cisplatin 70 $mg/m^2$

© 2000 Cancer Research Campaign and methotrexate 30 mg/m$^2$ on day 1 of a 21-day cycle has been investigated in patients refractory to at least one line of chemotherapy by Tu et al (Tu et al, 1995). In 25 patients an OR rate of 40% was observed but no CRs were reported.

We cannot be sure whether the lower response rate observed in our cohort of patients is due to schedule-dependent interactions between the three drugs or to different selection criteria (e.g. inclusion of patients who had received prior radiotherapy). Although we observed responses in locoregional disease others have observed major responses in all metastatic sites (Bajorin et al, 1998b) and this may simply have been a function of the smaller numbers in our series.

Toxicity observed with this regimen was predictable and generally manageable. All three drugs are neurotoxic and although 6 (40%) patients developed Grade 2 peripheral neuropathy only one patient progressed to Grade 3 neuropathy. The low incidence of severe peripheral neuropathy could be interpreted as further evidence of an antagonistic interaction between the three drugs when administered in this sequence and schedule.

We observed a lower than expected efficacy compared with either established regimens such as MVAC or the two-drug combination of paclitaxel and cisplatin. In addition there was less toxicity to the peripheral nerves than expected, which we suggest is consistent with the in vitro data for a schedule-dependent antagonistic interaction between vincristine and paclitaxel. Therefore this schedule of PVC cannot be recommended for further investigation. Exploration with more appropriate scheduling is necessary. However integration of paclitaxel and other new drugs such as gemcitabine with platinum compounds as already reported may hold more promise (Bellmunt et al, 1999; Vaishampayan et al, 1999).

### ACKNOWLEDGEMENTS

The Trial was organized jointly by the Medical Oncology Department at St. Bartholomew's Hospital and the Department of Clinical Oncology at the Velindre Hospital. Drug costs were supported by a grant from Bristol Myers Squibb Ltd.

### REFERENCES

Bajorin D, Dodd P, McCaffrey J, Mazumdar M, Vlamis V, Herr H, Boyle M, Scher H and Higgins G (1998a) M-VAC in transitional cell carcinoma (TCC): prognostic factors and long-term survival in 203 Patients (Pts). *Proceedings of ASCO* **17**: Abstr. 1198

Bajorin DF, McCaffrey JA, Hilton S, Mazumdar M, Kelly WK, Scher HI, Spicer J, Herr H and Higgins G (1998b) Treatment of patients with transitional-cell carcinoma of the urothelial tract with ifosfamide, paclitaxel, and cisplatin: a phase II trial. *Journal of Clinical Oncology* **16**: 2722–2727

Bellmunt J, Guillem V and Paz-Ares L et al (1999) A phase II trial of paclitaxel, cisplatin and gemcitabine (TCG) in patients (pts) with advanced transitional cell carcinoma (TCC) of the urothelium. *Proceedings of ASCO* **18**: Abstr. 1279

Blumenreich MS, Yagoda A, Natale RB and Watson RC (1982) Phase II trial of vinblastine sulfate for metastatic urothelial tract tumors. *Cancer* **50**: 435–438

Boshoff C, Oliver RT, Gallagher CJ and Ong J (1995) Accelerated cisplatin-based chemotherapy for advanced bladder cancer. *European Journal of Cancer* **31A**: 1633–1636

Burch P, Richardson R, Cha S, Sargent D, Pitot H, Kaur J and Camoriano J (1999) Phase II trial of combination paclitaxel and cisplatin in advanced urothelial carcinoma. *Proceedings of ASCO* **18**: Abstr. 1266

Dodd P, Mccaffrey J, Vlamis MM, Higgins G, Boyle M, Herr H, Scher H and Bajorin D (1998). Dose-intense M-Vac (Di-M-VAC) does not impact survival relative to standard M-VAC (M-VAC) in patients (Pts) with transitional cell carcinoma (TCC). *proceedings of ASCO* **17**: Abstr. 1223

Dreicer R, Roth B, Lipsitz S, Cohen M, See W and Wilding G (1998) E2895 Cisplatin And Paclitaxel in Advanced Carcinoma of the urothelium: a phase II trial of the Eastern cooperative oncology group (ECOG). *Proceedings of ASCO* **17**: Abstr. 1233

Giannakakou P, Villalba L, Li H, Poruchynsky M and Fojo T (1998) Combinations of paclitaxel and vinblastine and their effects on tubulin polymerization and cellular cytotoxicity: characterization of a synergistic schedule. *International Journal of Cancer* **75**: 57–63

Harker WG, Meyers FJ, Freiha FS, Palmer JM, Shortliffe LD, Hannigan JF, McWhirter KM and Torti FM (1985) Cisplatin, methotrexate, and vinblastine (CMV): an effective chemotherapy regimen for metastatic transitional cell carcinoma of the urinary tract. A Northern California Oncology Group study. *J Clin Oncol* **3**: 1463–1470

Hayward JL, Carbone PP, Heusen JC, Kumaoka S, Segaloff A and Rubens, RD (1977). Assessement of response to therapy in advanced breast cancer. *Br J Cancer* **35**: 292–298

Herr HW (1980). Cis-diamminedichloride platinum II in the treatment of advanced bladder cancer. *J Urol* **123**: 853–855

Jekunen AP, Christen RD, Shalinsky DR and Howell SB (1994). Synergistic interaction between cisplatin and Taxol in human ovarian carcinoma cells in vitro. *Br J Cancer* **69**: 299–306

Loehrer PJ, Sr., Einhorn LH, Elson PJ, Crawford ED, Kuebler P, Tannock I, Raghavan D, Stuart-Harris R, Sarosdy MF and Lowe BA and et al (1992). A randomized comparison of cisplatin alone or in combination with methotrexate, vinblastine, and doxorubicin in patients with metastatic urothelial carcinoma: a cooperative group study [published erratum appears in J Clin Oncol 1993 Feb; 11(2);384]. *J Clin Oncol* **10**: 1066–1073

Merrin C (1978) Treatment of advanced bladder cancer with cisdiamminedichloroplatinum (II NSC 119875): a pilot study. *J Urol* **119**: 493–495

Murphy B, Smith J, Koch M, DeVore R and Blanke C (1996) Phase II trial of paclitaxel (P) and cisplatin (C) for metastatic or locally unresectable urothelial Cancer. *Proceedings of ASCO* **15**: Abstr. 617

Natale RB, Yagoda A, Watson RC, Whitmore WF, Blumenreich M and Braun DW Jr (1981). Methotrexate: an active drug in bladder cancer. *Cancer* **47**: 1246–1250

Oliver RT, Newlands ES, Wiltshaw E and Malpas JS (1981). A phase 2 study of Cis-platinum in patients with recurrent bladder carcinoma. The London and Oxford Co-operative Urological Cancer Group. *Br J Urol* **53**: 444–447

Oliver RT, England HR, Risdon RA and Blandy JP (1984). Methotrexate in the treatment of metastatic and recurrent primary transitional cell carcinoma. *J Urol* **131**: 483–485

Papamichael D, Gallagher CJ, Oliver RT, Johnson PW and Waxman J (1997). Phase II study of paclitaxel in pretreated patients with locally advanced/metastatic cancer of the bladder and ureter. *British Journal of Cancer* **75**: 606–607

Parker SL, Tong T, Bolden S and Wingo PA (1997) Cancer statistics, 1997 [published erratum appears in CA Cancer J Clin 1997 Mar-Apr;47(2):68]. *Ca: Cancer Journal for Clinicians* **47**: 5–27

Peters PC and MR ON (1980). Cis-diamminedichloroplatinum as a therapeutic agent in metastatic transitional cell carcinoma. *J Urol* **123**: 375–377

Rossof AH, Coltman CA, Jr., Jones SE and Talley RW (1979). Phase II evaluation of cis-dichlorodiammineplatinum(II) in lymphomas: a Southwest Oncology Group Study. *Cancer Treat Rep* **63**: 1605–1608

Roth BJ, Dreicer R, Einhorn LH, Neuberg D, Johnson DH, Smith JL, Hudes GR, Schultz SM and Loehrer PJ (1994). Significant activity of paclitaxel in advanced transitional-cell carcinoma of the urothelium: a phase II trial of the Eastern Cooperative Oncology Group. *J Clin Oncol* **12**: 2264–2270

Rowinsky EK, Citardi MJ, Noe DA and Donehower RC (1993). Sequence-dependent cytotoxic effects due to combinations of cisplatin and the antimicrotubule agents taxol and vincristine. *J Cancer Res Clin Oncol* **119**: 727–733

Sternberg CN, Yagoda A, Scher HI, Watson RC, Ahmed T, Weiselberg LR, Geller N, Hollander PS, Herr HW and Sogani PC and et al (1985). Preliminary results of M-VAC (methotrexate, vinblastine, doxorubicin and cisplatin) for transitional cell carcinoma of the urothelium. *Journal of Urology* **133**: 403–407

Tu SM, Hossan E, Amato R, Kilbourn R and Logothetis CJ (1995). Paclitaxel, cisplatin and methotrexate combination chemotherapy is active in the treatment of refractory urothelial malignancies. *J Urol* **154**: 1719–1722

Vaishampayan U, Smith D, Redman B, Kucuk O, Ensley J and Hussain M (1999) Phase II evaluation of carboplatin, paclitaxel and gemcitabine in advanced urothelial carcinoma. *Proceedings of ASCO* **18**: Abstr. 1282

*© 2000 Cancer Research Campaign*

Yagoda A (1987) Chemotherapy of urothelial tract tumors. *Cancer* **60**: 574–585
Yagoda A, Watson RC, Gonzalez-Vitale JC, Grabstald H and Whitmore WF (1976). Cis-dichlorodiammineplatinum(II) in advanced bladder cancer. *Cancer Treat Rep* **60**: 917–923
Yagoda A, Watson RC, Whitmore WF, Grabstald H, Middleman MP and Krakoff IH (1977). Adriamycin in advanced urinary tract cancer: experience in 42 patients and review of the literature. *Cancer* **39**: 279–285

 *© 2000 Cancer Research Campaign*
